# CSF CXCL13 and Chitinase 3-like-1 Levels Predict Disease Course in Relapsing Multiple Sclerosis

**DOI:** 10.1007/s12035-022-03060-6

**Published:** 2022-10-10

**Authors:** Matteo Lucchini, Valeria De Arcangelis, Geny Piro, Viviana Nociti, Assunta Bianco, Chiara De Fino, Gabriele Di Sante, Francesco Ria, Paolo Calabresi, Massimiliano Mirabella

**Affiliations:** 1 Fondazione Policlinico Universitario Agostino Gemelli IRCCS, UOC Neurologia, Rome, Italy; 2 Centro Di Ricerca Sclerosi Multipla (CERSM), Università Cattolica del Sacro Cuore, Largo Agostino Gemelli 8, 00168 Rome, Italy; 3 Fondazione Policlinico Universitario Agostino Gemelli IRCCS, Oncologia Medica, Rome, Italy; 4 Dipartimento Di Medicina e Chirurgia, Sezione Di Anatomia Umana, Clinica e Forense, Università Degli Studi Di Perugia, Perugia, Italy; 5 Dipartimento Di Medicina E Chirurgia Traslazionale, Università Cattolica del Sacro Cuore, Rome, Italy; 6 Dipartimento Di Scienze Di Laboratorio Ed Infettivologiche, Fondazione Policlinico Universitario A. Gemelli IRCCS, Rome, Italy

**Keywords:** Multiple sclerosis, Biomarker, CSF, CNS, Chitinase 3-like1, BAFF, APRIL, Chemokine, CXC13, CXCL8, CXCL10, CXCL12, CCL2

## Abstract

Several biomarkers from multiple sclerosis (MS) patients’ biological fluids have been considered to support diagnosis, predict disease course, and evaluate treatment response. In this study, we assessed the CSF concentration of selected molecules implicated in the MS pathological process. To investigate the diagnostic and prognostic significance of CSF concentration of target candidate biomarkers in both relapsing (RMS, *n* = 107) and progressive (PMS, *n* = 18) MS patients and in other inflammatory (OIND, *n* = 10) and non-inflammatory (ONIND, *n* = 15) neurological disorders. We measured the CSF concentration of APRIL, BAFF, CHI3L1, CCL-2, CXCL-8, CXCL-10, CXCL-12, CXCL-13 through a Luminex Assay. MS patients were prospectively evaluated, and clinical and radiological activity were recorded. CHI3L1 and CXCL13 CSF levels were significantly higher in both MS groups compared to control groups, while CCL2, BAFF, and APRIL concentrations were lower in RMS patients compared to PMS and OIND. Considering RMS patients with a single demyelinating event, higher concentrations of CHI3L1, CXCL10, CXCL12, and CXCL13 were recorded in patients who converted to clinically defined MS(CDMS). RMS patients in the CXCL13 and CHI3L1 high concentration group had a significantly higher risk of relapse (HR 12.61 and 4.57), MRI activity (HR 7.04 and 2.46), and of any evidence of disease activity (HR 12.13 and 2.90) during follow-up. CSF CXCL13 and CHI3L1 levels represent very good prognostic biomarkers in RMS patients, and therefore can be helpful in the treatment choice. Higher CSF concentrations of neuro-inflammatory biomarkers were associated with a higher risk of conversion to CDMS in patients with a first clinical demyelinating event. Differential CSF BAFF and APRIL levels between RMS and PMS suggest a different modulation of B-cells pathways in the different phases of the disease.

**Supplementary Information:**

The online version contains supplementary material available at 10.1007/s12035-022-03060-6.

## Introduction

Multiple sclerosis (MS) represents the second commonest disabling disease in young adults after traumatic injuries with a significant and increasing impact on healthcare costs [1]. Although the underlying cause remains unknown, MS is classified as a chronic demyelinating inflammatory disorder of the central nervous system (CNS). The inflammatory process has been historically classified as a T-cell-mediated autoimmune pathology [2]. However, in the last years, the efficacy of B-cell-depleting therapies as well as novel pathological findings highlighted the involvement of the B-cell system[3].

MS patients can have a widely variable disease course from a highly active disease with rapid disability worsening to a long-term benign course. A prompt and precise diagnosis together with the recognition of risk factors for a worse prognosis are essential to establish a timely and effective treatment that may substantially modify the disease course. In this setting, the identification and validation of diagnostic and prognostic biomarkers from biological fluids can help the clinician in the therapeutic management of MS patients. Currently, the demonstration of CSF-specific oligoclonal bands (OCBs) represents the only laboratory test incorporated in the more recent revision of diagnostic criteria [4].

In this study, we analyzed the CSF concentration of selected molecules implicated in different MS pathological inflammatory pathways, involving B and T cells, and neurodegeneration. Included biomarkers were B-cell activating factor (BAFF); a proliferation-inducing ligand (APRIL); chitinase 3-like1 (CHI3L1); monocyte chemoattractant protein 1 (CCL2); chemokine ligand 8 (CXCL8, also named Interleukin 8), interferon gamma-induced protein 10 (CXCL10); Stromal cell–derived factor 1 (CXCL12); and B cell–attracting chemokine 1 (CXCL13).

APRIL and BAFF axis have been explored in MS pathology with evidence of BAFF hyperexpression in CNS of experimental autoimmune encephalomyelitis (EAE) model and upregulation in MS lesions [5, 6]. APRIL expression was demonstrated in lesions from EAE and post-mortem MS patients upon infiltration of macrophages [7]. Following these findings, drugs targeting these proteins have been tested in MS patients with negative results. Patients treated with atacicept showed an increase in disease activity with annualized relapse rate doubling [8] and negative data from the phase II tabalumab study were recently published [9].

CHI3L1 expression is induced by different inflammatory mediators and CHI3L1 can also increase the transcription levels of other pro-inflammatory molecules [10]. Activated microglia and reactive astrocytes are responsible for CHI3L1 production within the brain with CHI3L1 representing a biomarker of neuroinflammation and microglial activation. Those two phenomena are both particularly abundant in MS and relevant to its pathogenesis [11].

CCL2 represents a potent chemokine for monocytes and T cells [12] and its role in MS remains enigmatic as low levels are found in the CSF of patients while CCL2 is overexpressed in MS plaques, particularly in the active ones [13, 14]. It has been demonstrated that CCL2 is directly produced in the CNS and consumed by CCR2-positive migrating T cells and monocytes [15].

CXCL8 is a main pro-inflammatory chemokine which acts as a potent chemoattractant and activator of neutrophils and monocytes and regulates their adhesion to endothelial cells and migration across the vascular wall [16]. In MS, the CXCL8 receptor was detected on oligodendrocytes around active and silent lesions, and hypertrophic astrocytes stain strongly for CXCL8 in active MS lesions [17].

CXCL10 primarily acts as chemoattractant of macrophages, monocytes, and activated T and NK cells modulating T cell development and function. In MS active demyelinating lesions, CXCL10 was predominantly expressed by both macrophages (present inside the plaque) and reactive astrocytes in the surrounding parenchyma [18].

CXCL12 is extensively and constitutively expressed at a low level in the normal adult CNS. The expression level of CXCL12 is significantly increased within reactive astrocytes and endothelial cells in plaques of active human MS [19].

CXCL13 is a chemokine of the cellular B compartment, essential for the formation of lymphoid follicles in non-lymphatic organs (also demonstrated in MS) [5, 20, 21]. Regarding CSF CXCL13 concentration, the highest levels are present in patients with CNS infectious diseases. CSF CXCL13 elevation is, in fact, prominent in neuroborreliosis where it has been proposed as a diagnostic marker [22].

## Materials and Methods

### Study Design and Participants

This was an independent, monocentric, non-interventional study. We prospectively collected CSF and serum sample of MS patients attending the MS center of Fondazione Policlinico Universitario Agostino Gemelli IRCCS who underwent lumbar puncture for diagnostic purposes. Then, we retrospectively evaluated the clinical and radiological data of MS patients.

MS diagnosis was made according to the 2017 McDonald revised diagnostic criteria for relapsing MS (RMS) and Lublin criteria for progressive MS (PMS) [4, 23]. Neurological control group were classified as other non-inflammatory neurological diseases (ONIND) and other inflammatory neurological diseases (OIND) [24].

Inclusion criteria for MS patients were the following: diagnosis of MS following the most recent McDonald revised criteria; brain and spinal MRI within 30 days from CSF collection; at least 1-year prospective follow-up available; at least two brain and cervical and dorsal spinal MRI scans in the first year of follow-up and one further MRI scan yearly thereafter.

Patients were enrolled between April 2014 and July 2019. Follow-up was stopped in October 2020.

### CSF Storage and Analysis

The same sample collection procedure was applied to all patients. CSF was collected during the morning following standard procedure and then centrifuged for 10 min at 1000 RPM within 1 h from sample collection and stored at − 80° until further use [25].

All samples were analyzed for the presence of OCBs through CSF and serum immunoelectrophoresis. The presence of at least two bands in the CSF without correspondence in the serum was used to define the presence of OCBs [26]. OCBs have high sensitivity for MS diagnosis and are included in the 2017 McDonald diagnostic criteria [4]. We also evaluated the IgG index as for standard procedure (index between both serum and CSF IgG and albumin) [27]. An elevated IgG index is associated with MS diagnosis and represents both a risk factor for clinical conversion to MS in patient with a first demyelinating event and a long-term negative prognostic factor [28].

We evaluated the concentration of the following analytes through a Luminex Assay (R&D Systems, Minneapolis, USA): APRIL, BAFF, CHI3L1, CCL-2, CXCL-8, CXCL-10, CXCL-12, CXCL-13. We used a Luminex xMAP system (Bio-Plex 200 System, Bio-Rad Laboratories, CA) consisting of a multiplex biometric ELISA-based immunoassay containing dyed microspheres conjugated with a monoclonal antibody specific for a target protein [29]. CXCL-12 was evaluated in separate plates considering the peculiarities of this protein and the potential interference with other analytes reported by the manufacturer. We detected a value included in the standard curve from all samples for all biomarkers.

### Clinical and Radiological Outcomes

Age at CSF collection and sex were recorded for all patients.

For all MS patients, the following clinical and radiological data at baseline were collected: EDSS score performed by a certified neurologist (https://www.neurostatus.net/) [30]; multiple sclerosis severity score (MSSS) [31]; disease duration; presence of gadolinium-enhancing lesions (Gd +) and/or spinal cord lesions at baseline MRI scan.

Regarding RMS patients, we also recorded the number of previous relapses, the number of relapses in the year before CSF collection, and the eventual occurrence of clinical relapses 30 days within enrolment. A relapse was defined as any new neurological symptom, not associated with fever or infection, lasting for at least 24 h and accompanied by new neurological signs [4].

In patients with a history of a single demyelinating event (CIS/RMS), the occurrence of any new relapse during follow-up was used to define the conversion to clinically defined multiple sclerosis (CDMS) [32].

During follow-up, we recorded the occurrence of clinical or radiological disease activity (evidence of disease activity, EDA). The occurrence of any relapse during follow-up or the worsening of disability (defined as 1.5 point increase if baseline EDSS score was 0, 1.0 increase if baseline EDSS score was < 5.5, or 0.5 point increase if EDSS score was > 5.5, confirmed 6 months apart) was used to evaluate clinical disease activity [33]. The radiological activity was defined as the occurrence of Gd + lesions on T1-weighted images or new hyperintense lesions on T2-weighted images compared to the baseline scan.

For PMS patients, we calculated the progression index dividing the EDSS score by the disease duration expressed in years [34].

### Statistical Analysis

Continuous variables were described as mean ± standard deviation unless otherwise specified. Dichotomic or categorical variables were expressed as frequencies. Differences between RMS and PMS at baseline were explored with *t*-test for independent groups (for continuous variables) and chi-square test (for dichotomic and categorical variables) as appropriate.

The eventual correlation between ordinal variables at baseline and CSF biomarkers concentration was evaluated with Spearman’s rank correlation test. Comparisons of CSF biomarkers concentration between multiple groups were explored with ANOVA or Kruskal–Wallis test as appropriate. Comparisons between two independent groups were assessed through Student *T*-test and Mann–Whitney test, as appropriate. A normal distribution test (Kolmogorov–Smirnov test) and a test for the homogeneity of the variance (Levene test) were performed to guide the choice of parametric or non-parametric test.

Receiver operating characteristic (ROC) curves were fitted to estimate the diagnostic performance of CHI3L1 and CXCL13 CSF levels and to evaluate the ability of CHI3L1, CXCL10, CXCL12, and CXCL13 CSF concentrations in predicting the conversion to CDMS. The best diagnostic cut-off for these variables was determined with the Youden test. We performed a multivariate logistic regression analysis to combine the performance of CHI3L1, CXCL10, CXCL12, and CXCL13 CSF concentrations in predicting the conversion to CDMS.

To divide the RMS cohort based on CHI3L1 and CXCL13 CSF levels, we applied two different approaches: the first cut-off was derived from the previously described ROC analysis and Youden test, while the second one was calculated with a formula considering OIND values (mean ± 1.96 SD) exclusively [35].

Cox proportional hazards model was carried out to investigate the risk of disease activity stratified by CSF concentration of CHI3L1 and CXCL13.

All two-tailed *p*-values < 0.05 were considered as significant, without correction for multiple comparisons considering the exploratory study design. Data were analyzed by using the Statistical Package for Social Sciences, version 22.0(IBM SPSS, Inc., Chicago, Ill., USA).

## Results

### Study Population and Standard CSF Analysis

We enrolled 150 patients: 107 RMS, 18 PMS, 15 ONIND, and 10 OIND (Supplementary Table 1). Clinical and standard CSF analyses are reported in Table 1.

**Table 1:** Patients’ demographics

Patients *N* = 150	RMS *N* = 107	PMS *N* = 18	ONIND *N* = 15	OIND *N* = 10	*p* value
Female sex, *n* (%)	80 (74.8)	11 (61.1)	12 (80.0)	8 (80.0)	0.561
Age, years	37.4 (10.5)	52.9 (6.6)	41.3 (9.2)	47.6 (10.9)	< 0.001
IgG index	1.0 (0.7)	0.8 (0.5)	0.5 (0.1)	0.5 (0.2)	0.010
OCBs, *n* (%)	86 (80.4)	16 (88.9)	0 (0.0)	2 (20%)	< 0.001

All values are reported as mean (standard deviation) unless indicated otherwise. In bold are reported significant differences at a two-sided *α* level < 0.05. *RMS*, relapsing multiple sclerosis; *PMS*, progressive multiple sclerosis; *ONIND*, other non-inflammatory neurological diseases; *OIND*, other inflammatory neurological diseases; *OCBs*, oligoclonal bands.

The four different groups were homogeneous regarding sex with a female predominance. By contrast, we found a statistically significant difference regarding the age of patients. As expected, PMS patients were significantly older than RMS and ONIND.

Most patients in both MS groups had OCBs (80.4% and 88.9% respectively for RMS and PMS), whereas only two patients in the OIND group and none in the ONIND group presented OCBs.

Finally, the IgG index was significantly higher in MS compared with both control groups.

Clinical and radiological features of MS groups are summarized in Table 2.

**Table 2:** Clinical and radiological features of MS patients

MS patients *N* = 125	RMS *N* = 107	PMS *N* = 18	*p* value
FUP duration (years)	3.4 (1.4)	3.0 (1.5)	0.271
Disease duration (months), median [IQR]	4 [2–19]	44.5 [34.5–80.5]	0.044
EDSS, median [IQR]	1.5 [1.0–2.0]	4.0 [3.4–4.3]	< 0.001
Gd + lesion at baseline, *n* (%)	53 (52.5)	5 (27.8)	0.143
Spinal lesion at baseline, *n* (%)	84 (78.5)	17 (94.4)	0.193
MSSS	4.2 (2.1)	7.2 (1.2)	< 0.001
Previous relapses, median [range]	1 [1–4]	NA	
Relapses one-year preceding CSF collection	0.9 (0.4)	NA	
Relapse 30 days preceding CSF collection, *n* (%)	36 (33.6)	NA	
Progression index	NA	1.2 (0.8)	

All values are reported as mean (standard deviation) unless indicated otherwise. In bold are reported significant differences at a two-sided α level < 0.05. *MS*, multiple sclerosis; *RMS*, relapsing multiple sclerosis; *PMS*, progressive multiple sclerosis; *FUP*, follow-up; *IQR*, interquartile range; *EDSS*, expanded disability status scale; *Gd* + , gadolinium-enhancing; *MSSS*, multiple sclerosis severity score; *CSF*, cerebrospinal fluid.

Focusing on the RMS group, the mean follow-up duration was 3.4 years (SD 1.4) while the median disease duration was 4 months (IQR 2–19). The median EDSS score was 1.5. Interestingly, most patients were evaluated after their first clinical relapse (88 CIS/RMS). Nearly half of RMS patients had at least one Gd + lesion while 78.5% had at least one spinal lesion at baseline scan. Thirty-six (33.6%) had a clinical relapse within 30 days from CSF collection.

All patients were not exposed to any disease-modifying therapies (DMTs) before CSF analysis. Only eight patients (7.5%) did not start a DMT during follow-up. In the other patients, a DMT was initiated within 3 months from CSF collection. Most patients started a first-line DMT while a highly effective DMTs was the first therapy in 16 cases (14.9%; 10 natalizumab, 4 ocrelizumab, and 2 alemtuzumab). This group included two patients with two relapses in the year before enrolment and 12 patients with an early relapse (within 90 days from CSF collection) (Supplementary Table 2).

In the PMS group, median EDSS was 4.0. Almost all patients (94.4%) had at least one spinal lesion, while 5 (27.8%) patients had Gd + lesions at baseline scan. In this subgroup, we evaluated the eventual correlation between CSF biomarkers and demographic data such as age and sex in PMS patients, but we did not find any significant difference. Next, we explored potential correlation with disease severity indices such as EDSS score, MSSS score, and progression index (PI). Considering the low sample size of the PMS cohort, we reported only data for statistically significant strong correlation (*r* coefficient > 0.7). We found a significant strong positive correlation between CXCL10 level and MSSS (Spearman correlation *r* 0.74, *p*-value < 0.01; while CXCL10 level and PI Spearman correlation was 0.61) (Supplementary Fig. 1).

The low sample size of the PMS group, together with a variable therapeutic approach (ocrelizumab treatment introduced in 2017) prevented further analysis.

### Biomarkers Levels: Comparison Between MS and Control Groups

The concentration levels of analyzed biomarkers are reported in Table 3 (expressed as pg/ml for all proteins except for CHI3L1 measured as ng/ml).

**Table 3:** CSF biomarkers concentrations

Patients *N* = 150	RMS *N* = 107	PMS *N* = 18	ONIND *N* = 15	OIND *N* = 10	*p* value
APRIL	60.4 (130.2) 58.7 [36.7–85.3]	95.5 (37.7) 94.8 [63.7–125.6]	69.0 (30.9) 64.7 [40.9–88.5]	71.1 (31.7) 65.9 [48.9–101.6]	0.003
BAFF	133.4 (72.8) 120.8 [85.6–163.5]	175.6 (61.6) 164.4 [114.3–229.0]	169.6 (62.3) 159.4 [123.6–199.4]	184.1 (53.1) 166.5 [150.2–238.6]	0.013
Chitinase 3 like 1, ng/ml	312.8 (436.9) 152.4 [96–253]	270.6 (161.4) 213.2 [154.6–342.7]	94.5 (39.3) 83.3 [75.4–105.5]	168.8 (77.1) 138.2 [123.8–217.0]	< 0.001*
CCL2	282.0 (130.2) 266.9 [201.2–353.3]	388.5 (178.2) 379.3 [262.2–447.1]	369.3 (114.7) 346.5 [266.7–461.9]	314.0 (54.7) 339.6 [249.1–359.3]	0.004
CXCL8	38.9 (49.3) 30.8 [24.3–40.2]	41.1 (15.3) 39.2 [29.8–53.0]	28.0 (8.8) 25.3 [22.2–33.7]	27.8 (7.4) 26.5 [21.4–33.9]	0.676
CXCL10	78.3 (86.0) 58.1 [36.9–91.9]	92.0 (61.6) 74.2 [52.6–122.2]	44.8 (22.8) 37.3 [30.7–60.6]	61.1 (44.0) 51.4 [27.3–75.6]	0.305
CXCL12	621.9 (366.5) 585.0 [372.6–819.2]	801.1 (463.2) 852.4 [396.3–1176.8]	691.2 (438.8) 538.5 [429.4–789.4]	676.3 (361.6) 697.5 [386.7–963.3]	0.321
CXCL13	19.8 (28.5) 11.5 [8.6–19.9]	12.7 (5.8) 11.3 [9.6–15.0]	6.9 (3.1) 6.7 [4.9–9.0]	11.1 (6.6) 8.0 [6.3–17.0]	< 0.001*

All values are reported as mean (standard deviation) in the first row and median [interquartile range] in the second row. In bold are reported significant differences at a two-sided *α* level < 0.05. A single asterisk (*) for non-parametric tests. *CSF*, cerebrospinal fluid.

**Fig. 1:**
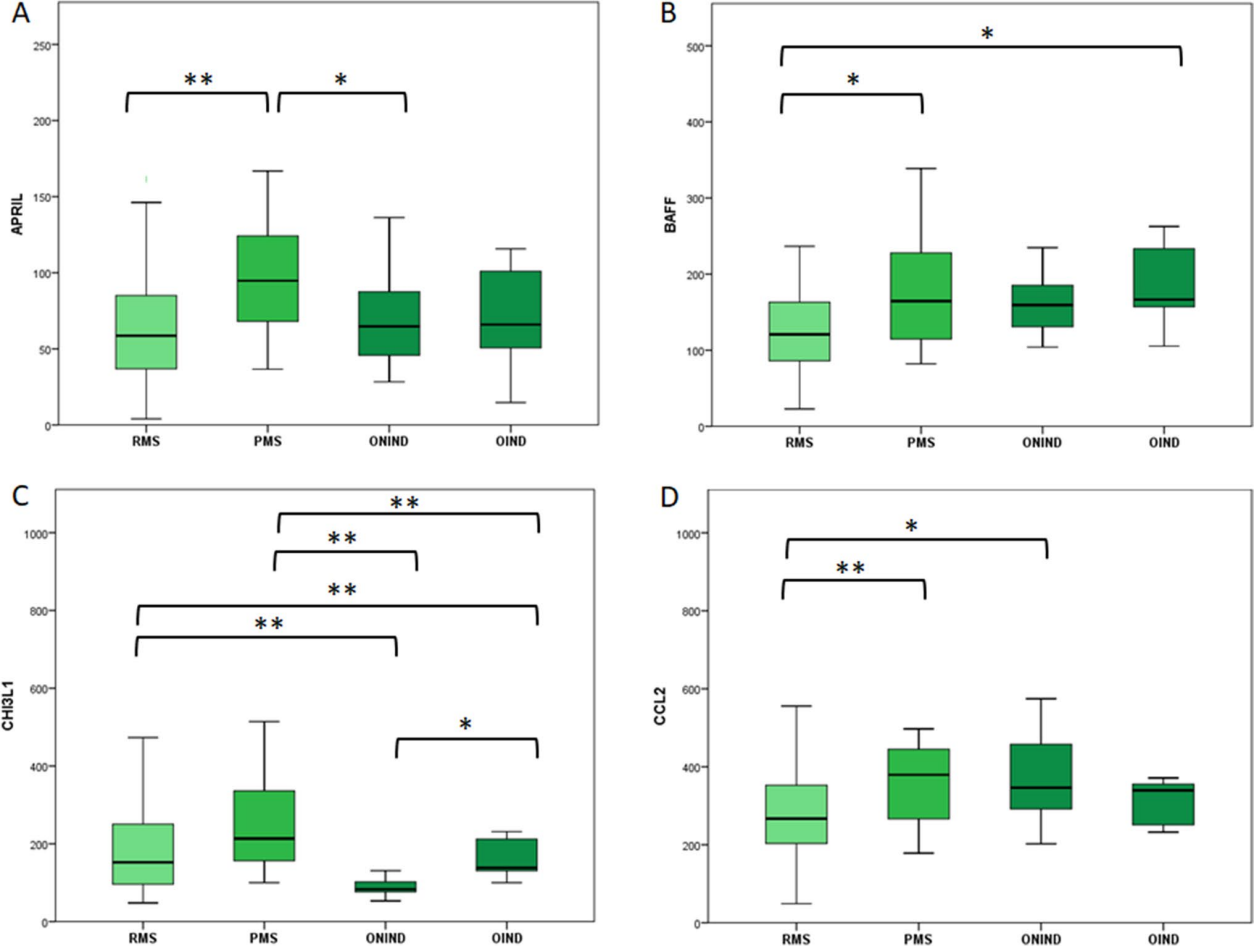
CSF concentration of APRIL (**A**), BAFF (**B**), CHI3L1 (**C**), and CCL2 (**D**) for each study group in pg/ml except for CHI3L1 measured as ng/ml. The boxes represent median and interquartile range. Statistical differences between groups were highlighted: a singler asteris (*) for *p*-value < 0.05 and double asterisks (**) for *p*-value < 0.01. Comparisons of CSF biomarkers concentration between multiple groups were explored with ANOVA for APRIL, BAFF, and CCL-2 and with Kruskal–Wallis for CHI3L1. Comparisons between two independent groups were assessed through Student *T*-test for APRIL, BAFF, and CCL-2 and with Mann–Whitney test for CHI3L1. RMS, relapsing multiple sclerosis; PMS, progressive multiple sclerosis; ONIND, other non-inflammatory neurological disorders; OIND, other inflammatory neurological disorders

Regarding APRIL and BAFF, we found statically significant differences between the four subgroups (*p* = 0.003 and 0.013 respectively, ANOVA test). (Fig. 1A, B).

RMS presented significantly lower concentrations of both APRIL and BAFF when compared to PMS (*p* < 0.001 and 0.024 respectively). BAFF concentrations were also lower in RMS compared to control groups reaching statistical significance only with OIND (*p* = 0.069 vs. ONIND and *p* = 0.034 vs. OIND). We did not find significant differences between RMS and control groups regarding APRIL concentrations.

BAFF concentrations did not differ significantly between PMS and both control groups. whereas APRIL concentrations were higher in PMS respect to ONIND (*p* = 0.037) and OIND (*p* = 0.096).

CHI3L1 CSF concentrations were significantly higher in both MS groups in comparison to both control groups (Fig. 1C). We did not find significant differences between RMS and PMS while CHI3L1 concentrations were significantly higher in OIND respect to ONIND (*p* = 0.016).

Considering the significant differences between MS and both control groups, we performed ROC curve analysis to evaluate the diagnostic power of CHI3L1. Area under the curve (AUC) was 0.69 (IC 95% 0.59–0.79, p = 0.003) considering both control group and raised to 0.80 (IC 95% 0.70–0.89, *p* =  < 0.001) after excluding OIND group. Including all control patients, a diagnostic cut-off of 148.0 ng/ml was identified through Youden test with a sensitivity of 56.0% and a specificity of 84.0%. After excluding OIND patients, we identified a lower cut-off (113.4 ng/ml) with 70.4% sensitivity and 86.7% specificity (Supplementary Fig. 1A-B).

CSF CCL2 concentration was significantly lower in RMS patients respect to PMS and ONIND while PMS have comparable concentrations with both control groups. (Fig. 1D).

**Fig. 2:**
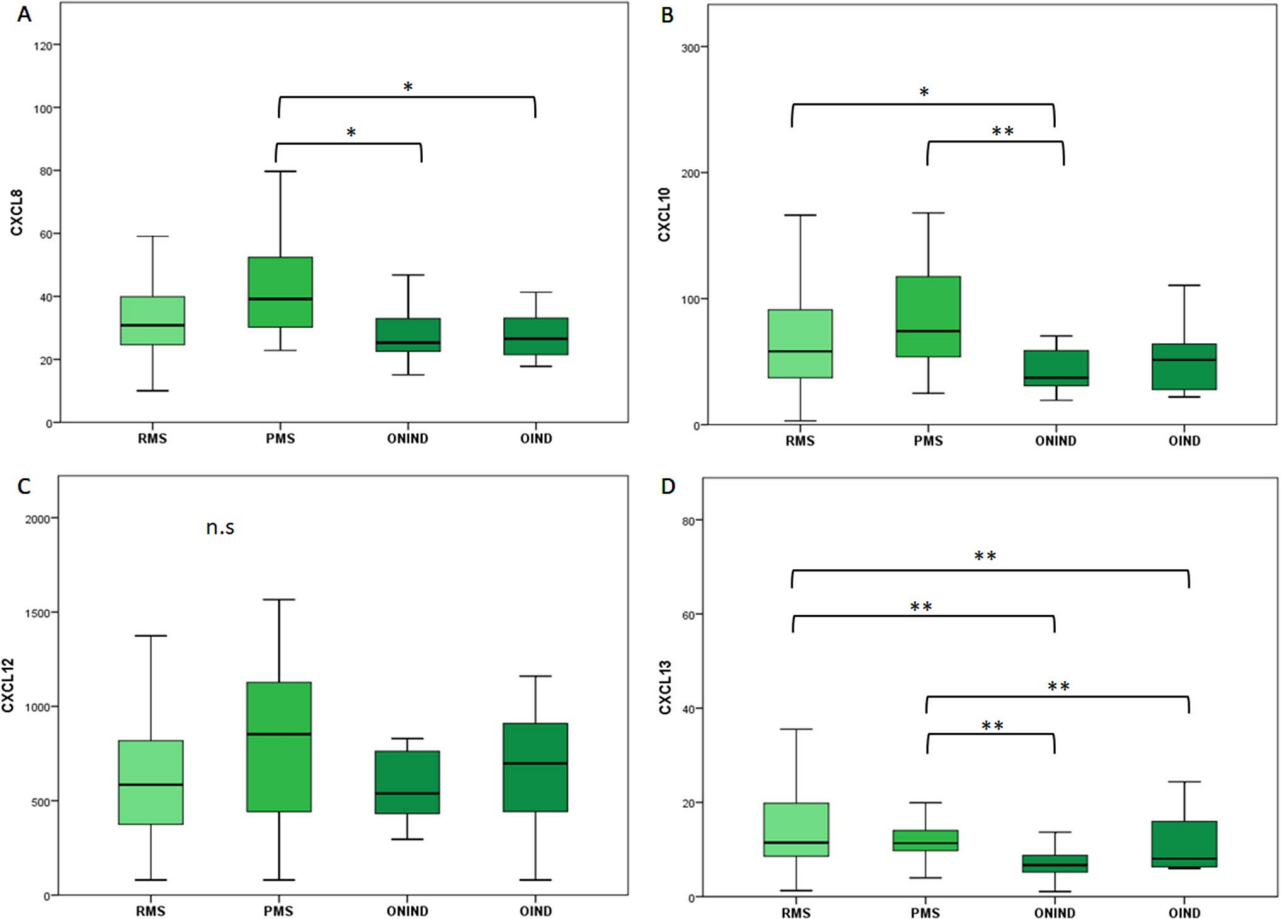
CSF concentration of CXCL8 (**A)**, CXCL10 (**B**), CXCL12 (**C**), and CXCL13 (**D**) for each study group in pg/ml except for CHI3L1 measured as ng/ml. The boxes represent median and interquartile range. Statistical differences between groups were highlighted: a singler asteris (*) for *p*-value < 0.05 and double asterisks (**) for *p*-value < 0.01. Comparisons of CSF biomarkers concentration between multiple groups were explored with ANOVA for CXCL8, CXCL10, and CXCL12 and with Kruskal–Wallis for CXCL13. Comparisons between two independent groups were assessed through Student *T*-test for CXCL8, CXCL10, and CXCL12 with Mann–Whitney test for CXCL13. RMS, relapsing multiple sclerosis; PMS, progressive multiple sclerosis; ONIND, other non-inflammatory neurological disorders; OIND, other inflammatory neurological disorders

We did not find significant differences between groups through ANOVA test for CXCL8, CXCL10, and CXCL12 (Fig. 2A-C).

Despite this, we found significantly higher CSF CXCL10 levels both in RMS and PMS when compared to OIND (*p* = 0.038 and 0.002 for RMS and PMS, respectively).

We also found that mean CXCL8 levels were higher in MS patients compared to OIND and ONIND. These differences were statistically significant only for the PMS group.

CSF CXCL13 concentration was significantly higher in MS when compared to control groups (*p* < 0.001). We did not find significant differences between OIND and ONIND (Fig. 2D).

Then, we performed ROC curve analysis to evaluate the diagnostic power of CXCL13. AUC was 0.77 (IC 95% 0.66–0.87, *p* < 0.001) considering both control group and raised to 0.83 (IC 95% 0.74–0.93, *p* < 0.001) after excluding OIND group. Including all control patients, a diagnostic cut-off of 8.9 pg/ml was identified through the Youden test with a sensitivity of 74.4% and a specificity of 76.0%. After excluding OIND patients, we identified a higher cut-off (10.1 pg/ml) with 60.0% sensitivity and 93.3% specificity (Supplementary Fig. 2C-D).

### Disease Activity in RMS Patients During Follow-up

After a median follow-up of 3.4 years (IQR 2.2-–4.7 years), 35 (32.7%) patients had at least one relapse while follow-up MRI scans showed new radiological activity in 55 (51.4%). Only 8 (7.5%) patients had disability progression. Altogether, 62 (57.9%) patients had clinical and/or radiological activity during follow-up. None of the patients included in the RMS cohort transitioned to a progressive disease course during the study period.

We then evaluated the potential relationship between explored CSF biomarkers and demographic, clinical, and radiological features. We found a significantly higher concentration of CXCL12 and CXCL13 in male patients (*p* < 0.01) (data not shown). We evaluated eventual differences in the biomarkers’ concentration between patients with or without recent clinical or radiological activity. We did not find any statically significant difference except for CXCL12 concentration that resulted slightly higher in patients with a recent relapse (*p* = 0.055; 569.1 pg/ml vs. 725.9 pg/ml). CXCL13 and CHI3L1 levels both presented a weak positively correlation with EDSS (Spearman correlation, *r* 0.26 and 0.28, *p* < 0.01) and MSSS score (*r* 0.370 and 0.264, *p* < 0.01) (data not shown). We did not find significant differences regarding the presence of OCBs or imaging features such as Gd + or spinal lesion at baseline MRI.

### Predictors of Conversion to CDMS in CIS/RMS Patients

Eighty-eight patients were evaluated after their first demyelinating event. During follow-up, 29 patients experienced a new clinical relapse, thus converting to CDMS. Only eight patients were not treated with DMTs during follow-up since they did not present further clinical or radiological activity.

**Fig. 3:**
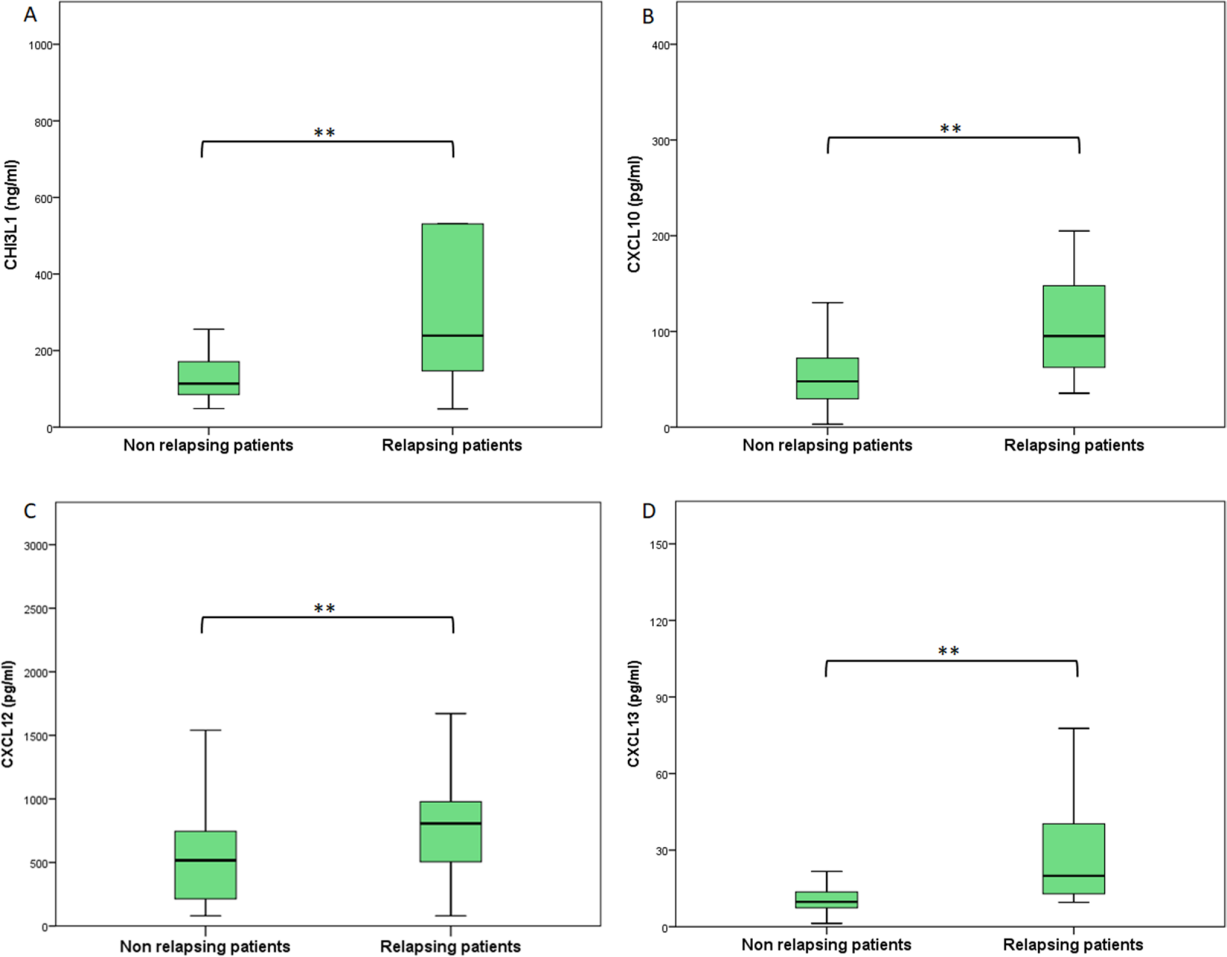
CSF concentration of CHI3L1 (**A**), CXCL10 (**B**), CXCL12 (**C**), CXCL13 (**D**) in patients with a single demyelinating event at baseline. The two groups were divided following the occurrence (relapsing) or the absence (non-relapsing) of new relapse. The boxes represent median and interquartile range. Statistical differences (Student *t*-test for CXCL12 and Mann–Whitney test for CHI3L1, CXCL12, and CXCL13) between groups were highlighted: a singler asteris (*) for *p*-value < 0.05 and double asterisks (**) for *p*-value < 0.01

In this cohort, we evaluated the eventual differences in CSF biomarkers concentrations between converting and non-converting patients. Patients who converted to CDMS had significantly higher CSF concentrations of CHI3L1, CXCL10, CXCL12, and CXCL13 compared to non-converting patients (Fig. 3A-D and Supplementary Table 3).

**Fig. 4:**
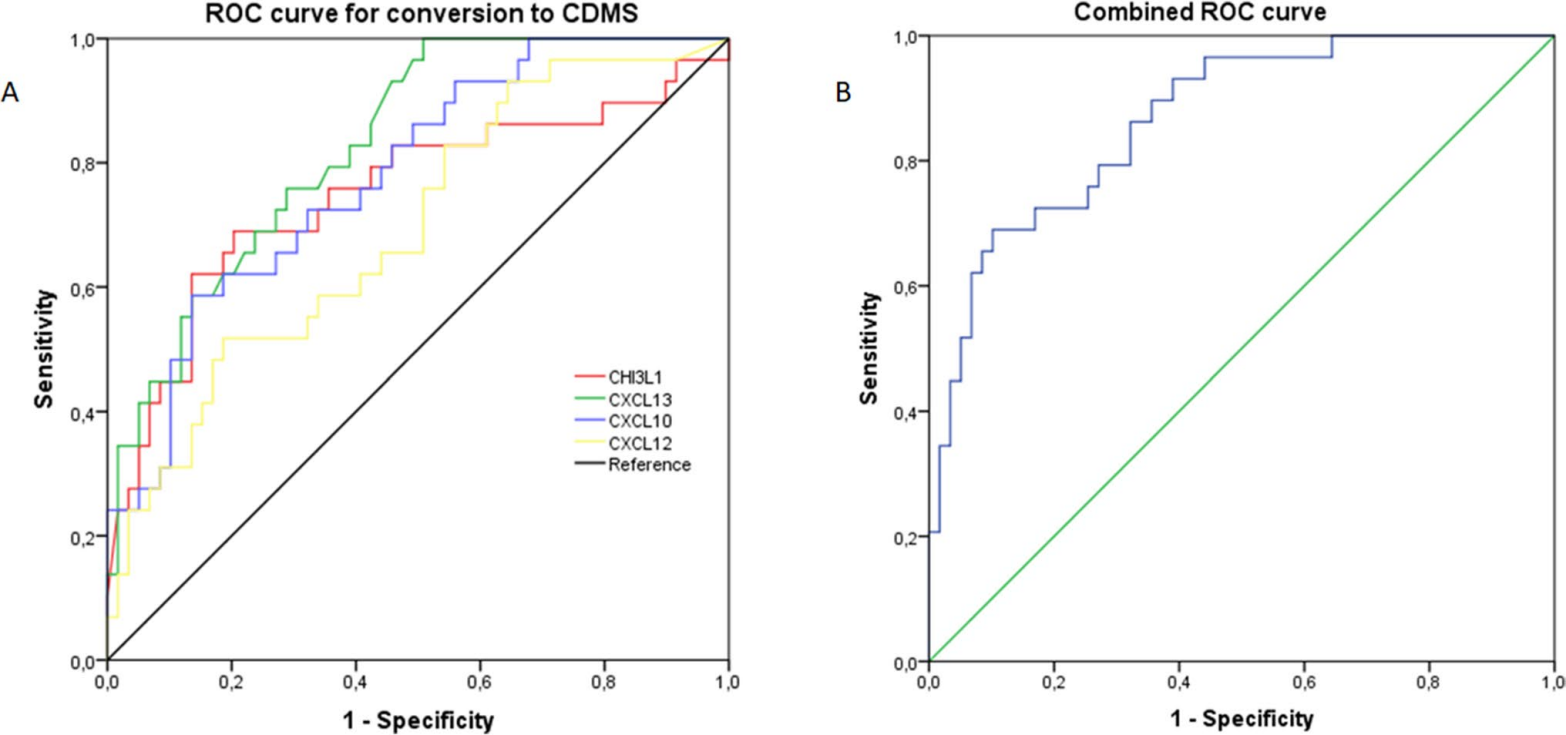
ROC curve to predict conversion to CDMS in patients with a first demyelinating event at baseline. **A** Each line represents the ROC curve for a single CSF biomarker (CHI3L1, CXCL13, CXCL2, and CXCL10). **B** ROC curve obtained through a multivariate regression analysis including biomarkers associated with the risk of conversion to CDMS. *p* < 0.01 for all analysis. ROC, receiver operating characteristic; CDMS, clinically defined multiple sclerosis

ROC curves for these biomarkers were all statistically significant with the following AUC (descending order): CXCL13 0.83 (IC 95% 0.74–0.91, *p* < 0.001), CXCL10 0.78 (IC 95% 0.68–0.88, *p* < 0.001), CHI3L1 0.75 (IC 95% 0.63–0.87, *p* < 0.001), and CXCL12 0.69 (IC 95% 0.58–0.81, *p* 0.003).(Fig. 4A).

We also performed a multivariate logistic regression analysis to evaluate the predictive performance of the combination of the four CSF biomarkers with a statistically significant different concentration between converting and non-converting CDMS patients. The resulting AUC of ROC curve was 0.87 (IC 95% 0.79–0.94, *p* < 0.001) (Fig. 4B).

### Predictors of Clinical and Radiological Activities in RMS Patients

We next explored whether selected biomarkers could predict subsequent disease activity. Following our results, we selected CHI3L1 and CXCL13. Their concentration resulted significantly higher than both control groups and these two biomarkers had the highest AUC values at ROC curve analysis to distinguish MS patients from controls (Supplementary Fig. 2).

We then divided our cohort into three groups based on CHI3L1 and CXCL13 CSF concentration (low, intermediate, and high). The first cut-off was chosen based on ROC analysis and the Youden test as previously described. This cut-off divided patients into low and intermediate concentration groups. To separate intermediate and high concentration, we subsequently selected a higher cut-off based on biomarker concentration in the OIND group with this formula: mean OIND concentration + 1.96 SD.

**Fig. 5:**
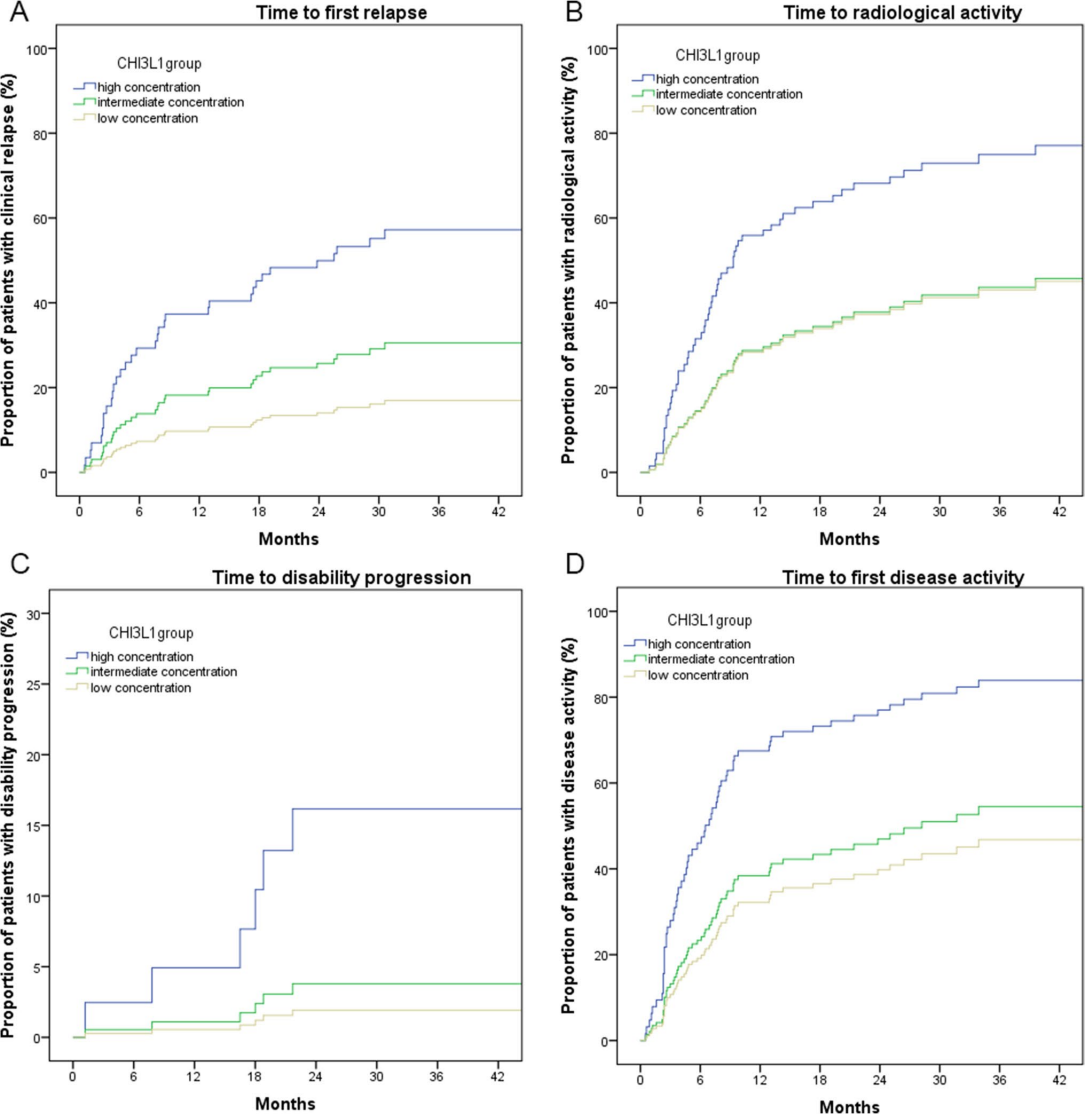
Cox regression analysis to represent the proportion of patients with disease activity (relapse in **A**; MRI activity in **B**; disability progression in **C**; any disease activity in **D**) based on the CSF concentration of CHI3L1. MRI, magnetic resonance imaging; CSF, cerebrospinal fluid

Focusing on CHI3L1, the two cut-offs were 111.9 and 319.9 ng/ml. The demographic, clinical, and radiological features of the three groups are reported in Supplementary Table 4. By comparing these groups, we found a significant difference for EDSS and MSSS score (higher scores in CHI3L1 high concentration group). We then used these variables as covariates in our Cox regression analysis to prevent the potential confounding effect of EDSS and MSSS scores. Through a Cox regression analysis, we found that patients in the CHI3L1 high concentration group had a significantly higher risk of subsequent relapse (HR 4.57), radiological activity (HR 2.46), and disease progression (HR 9.08) (Fig. 5A-D) (Table 4).

**Table 4:** Cox regression models to evaluate the risk of disease activity based on CHI3L1 CSF concentrations

	HR	95% CIs	*p*
Time to first relapse			
CHI3L1 low	1.0		Ref
CHI3L1 intermediate	1.96	0.73–5.26	0.180
CHI3L1 high	4.57	1.73–12.10	0.002
Time to first MRI activity			
CHI3L1 low	1.0		Ref
CHI3L1 intermediate	1.02	0.51–2.03	0.957
CHI3L1 high	2.46	1.26–4.80	0.008
Time to disability progression			
CHI3L1 low	1.0		Ref
CHI3L1 intermediate	1.99	0.18–22.0	0.574
CHI3L1 high	9.08	1.03–80.3	0.047
Time to first disease activity			
CHI3L1 low	1.0		Ref
CHI3L1 intermediate	1.25	0.66–2.38	0.500
CHI3L1 high	2.90	1.52–5.52	0.001

*HR*, hazard ratio; *95% CIs*, 95% confidence intervals. In bold are reported significant differences at a two-sided *α* level < 0.05. *CHI3L1*, chitinase 3-like1; *CSF*, cerebrospinal fluid.

**Fig. 6:**
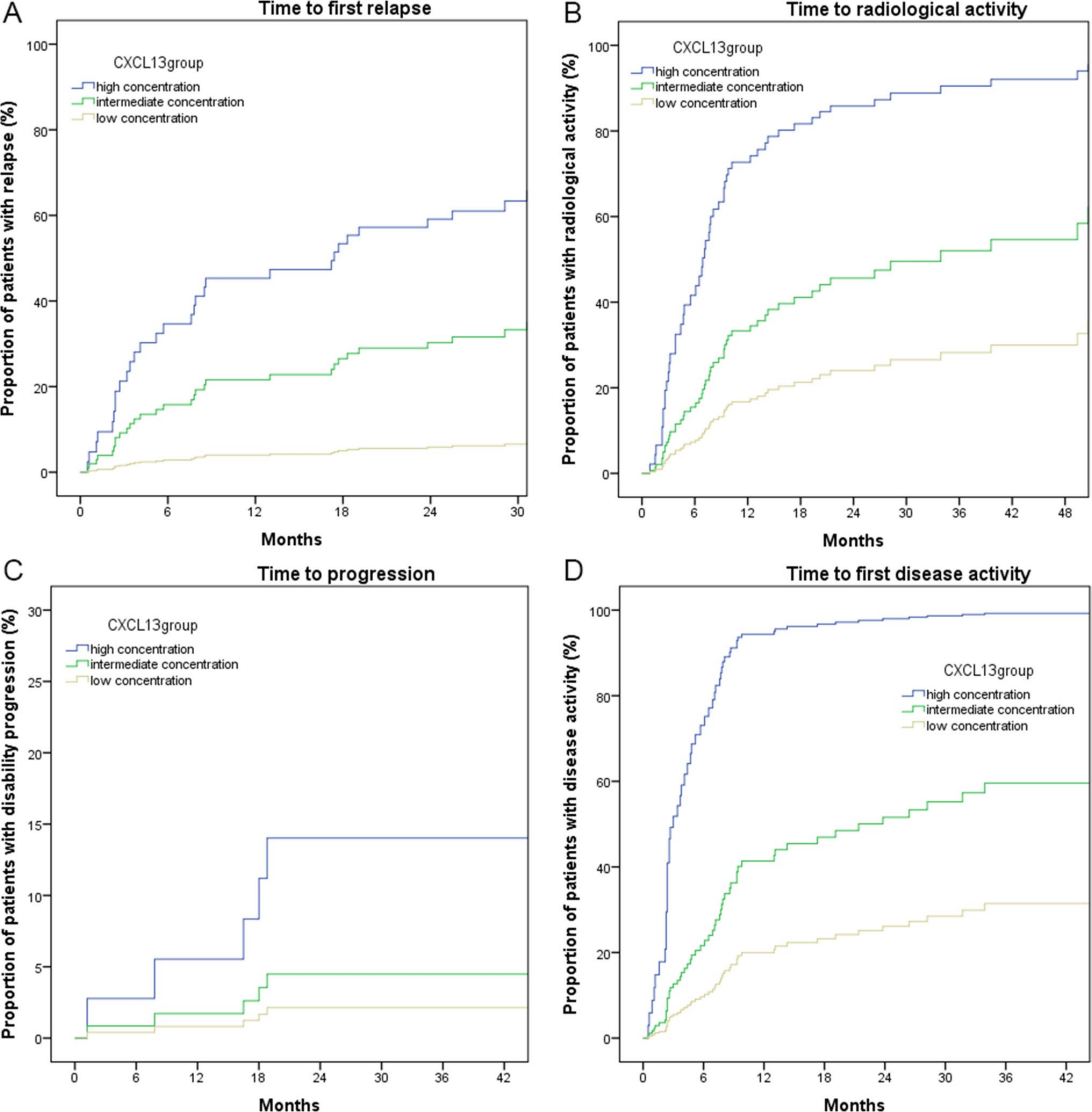
Cox regression analysis to represent the proportion of patients with disease activity (relapse in **A**; MRI activity in **B**; disability progression in **C**; any disease activity in **D**) based on the CSF concentration of CXCL13. MRI, magnetic resonance imaging; CSF, cerebrospinal fluid

Regarding CXCL13, we used the following cut-offs to divide RMS cohort: 8.9 pg/ml and 24.0 pg/ml respectively. We found a statistically significant difference between these groups for sex, MSSS score, the annualized relapse rate in the year before CSF collection, and the proportion of patients with Gd + lesion at baseline scan (Supplementary Table 5). These variables were consequently included in our regression analysis as previously described for CHI3L1. We found an increased risk of new relapse (HR 12.61), new radiological activity (HR 7.04), and EDA (HR 12.13) in both high and intermediate concentration groups when compared to the low concentration group. On the contrary, we did not find any differences regarding time to disability progression (Fig. 6A-D) (Table 5).

**Table 5:** Cox regression models to evaluate the risk of disease activity based on CXCL13 CSF concentrations

	HR	95% CIs	*P*
Time to first relapse			
CXCL13 low	1.0		Ref
CXCL13 intermediate	5.13	1.16–22.82	0.032
CXCL13 high	12.61	2.53–62.84	0.002
Time to first MRI activity			
CXCL13 low	1.0		Ref
CXCL13 intermediate	2.29	1.01–5.22	0.049
CXCL13 high	7.04	2.75–18.00	< 0.001
Time to disability progression			
CXCL13 low	1.0		Ref
CXCL13 intermediate	2.13	0.19–23.43	0.536
CXCL13 high	7.02	0.63–78.31	0.113
Time to first disease activity			
CXCL13 low	1.0		Ref
CXCL13 intermediate	2.20	1.01–4.83	0.049
CXCL13 high	12.13	4.89–30.08	< 0.001

*HR*, hazard ratio; *95% CIs*, 95% confidence intervals. In bold are reported significant differences at a two-sided *α* level < 0.05. *CSF*, cerebrospinal fluid.

## Discussion

Despite the demonstration of a complex immune system dysregulation, MS pathogenesis remains not fully understood. MS was firstly thought and described as a T-cell-mediated pathology but there are growing data pointing to a concomitant and significant B-cells involvement [2, 3].

In our study, we evaluated the CSF concentration of selected inflammatory biomarkers in a large group of MS patients, including both RMS and PMS, as well as in two control groups represented by OIND and ONIND. Biomarkers were selected based on their known involvement in MS-specific pathological processes both in vitro and in vivo.

By comparing MS and control groups, we found higher CSF levels of CHI3L1, CXCL13 in both MS groups compared to both inflammatory and non-inflammatory controls. CSF CHI3L1 levels were able to discriminate MS from non-inflammatory controls with 70.4% sensitivity and 86.7% specificity. Although less sensitive, CXCL13 CSF concentration showed very high specificity (60.0% sensitivity and 93.3% specificity). Several studies have also explored CSF CXCL13 expression in MS patients showing higher levels than in healthy controls or in patients with other inflammatory neurological diseases [36, 37].

Furthermore, CXCL10 levels were higher in both RMS and PMS compared to non-inflammatory controls and CXCL8 levels in PMS compared to both control groups. CSF CXCL8 concentration has been studied in MS and other CNS inflammatory disorders showing higher levels in antibody-mediated diseases such as neuromyelitis optica rather than in MS [38]. One recent paper has also proposed higher CSF CXCL8 levels in RMS as medium-term negative prognostic factor [39].

We additionally evaluated the CSF level of BAFF and APRIL. We found lower CSF concentration of both proteins in the RMS group compared to other groups while PMS patients presented the highest CSF APRIL levels.

CSF levels of BAFF and APRIL in RMS patients were evaluated without conclusive data. Most investigations suggest that CSF BAFF and APRIL levels were lower or comparable to non-inflammatory control groups. Piazza et al. firstly reported a lower concentration of both BAFF and APRIL in MS patients compared to different inflammatory and non-inflammatory neurological disorders [40]. This finding was confirmed in a few subsequent studies [35, 41, 42]. Moreover, two studies found that CSF BAFF levels were significantly lower in MS patients with OCBs compared to those without OCBs [29, 43]. By contrast, one paper described elevated CSF BAFF levels during relapse and another one showed increased levels of CSF APRIL and BAFF in MS patients with higher levels of gray matter damage at diagnosis [44, 45].

In our study, we did find a differential pattern of CSF BAFF and APRIL expression between RMS (lower levels) and PMS (higher levels). A possible interpretation is, on one hand, that after been produced and released by astrocytes, BAFF and APRIL are consumed by local plasma cells during early active phase of the disease with a consequent decrease of CSF levels of these cytokines [7]. On the other hand, a more diffuse astroglial proliferation, together with a decreased burden of inflammation, could be responsible for BAFF and APRIL upregulation in the progressive phase of the disease [46, 47].

In the second part of our study, we focused on the demonstration of a potential predictive value of such biomarkers in RMS patients.

The progressive evolution of diagnostic criteria led to very early diagnosis with a significant increase of patients being diagnosed as MS at the time of the first clinical demyelinating event. In our study cohort, we included 88 CIS/RMS patients. We found that patients who converted to CDMS had significantly higher CSF levels of CHI3L1, CXCL10, CXCL12, and CXCL13. Moreover, CSF levels of these biomarkers could accurately discriminate CDMS patients from non-converting patients (combined AUC 87%).

CHI3L1 as potential biomarkers of clinical conversion in CIS patients was firstly explored by Comabella et al. in two Spanish independent CIS cohorts and later confirmed in another study from a French cohort [48, 49]. In our previous paper, we found that CIS patients who converted to 2010 McDonald MS had higher CSF CHI3L1 compared to non-converting patients [29]. In the present study, we chose an even higher outcome (CDMS) that better reflects significant disease activity. In fact, although MRI activity represents an extremely valid surrogate of disease activity, the weight of a single relapse overwhelms isolated radiological activity in terms of long-term prognosis [50, 51]. Regarding CXCL13, a few studies found that higher CSF levels in CIS patients were associated with the risk of fulfilling McDonald criteria for RMS while one study demonstrated a high risk of conversion to CDMS in patients with optic neuritis [52–54].

Finally, through a proportional Cox regression analysis, we explored the predictive value of CSF CXCL13 and CHI3L1 levels in RMS regarding the future occurrence of disease activity, evaluated as clinical relapse, MRI activity, and disability progression. We here demonstrated that patients in the high concentration group of both CXCL13 and CHI3L1 had a significantly higher risk of relapse, MRI activity, and of EDA. Although disability progression had a low incidence (7.5%), the CHI3L1 high concentration group had also a significantly higher risk of disability progression. Two previous studies, one on RMS and the other on PMS, similarly found an increased risk of EDSS worsening in patients with higher CSF CHI3L1 levels [55, 56].

Although a reduction of CXCL13 was demonstrated after DMT treatment in MS patients, some patients still displayed increased CXCL13 CSF levels possibly identifying a subgroup with poorer prognosis [57–59].

Our data suggest that, among the candidate CSF biomarkers examined, CHI3L1 and CXCL13 can predict disease activity in RMS patients and help to identify patients with more severe disease course independently from other baseline clinical and radiological features. Late DMT initiation is recognized as a strong predictor of poorer long-term outcome; likewise, late switch to more effective DMTs after disease activity [60–62]. Moreover, there is growing evidence that highly active patients strongly benefit from directly starting treatment with highly effective DMTs such as monoclonal antibodies [63].

Furthermore, it should be underlined that our data have been obtained in a large RMS cohort with a very short disease duration at the time of CSF collection (78.3% with no more than 12 months clinical history) and a significant mean follow-up duration. In this context, the identification at baseline of independent risk factors for succeeding disease activity such as very high CSF CHI3L1 and CXCL13 levels could have a significant impact on treatment decisions and consequently on patients’ prognosis.

### Study Limits

Despite most patients started a fist-line DMT, in our cohort few patients remained untreated or, on the opposite, were directly exposed to highly effective DMTs. This aspect represents a partial limit of our study considering that untreated patients were clinically and radiologically stable while patients exposed to highly effective DMT had an early clinical and radiological activity included in the analysis. Some patients who experienced clinical and radiological activity during follow-up switched to highly effective DMTs. Nevertheless, this last aspect did not influence our prognostic evaluation since those patients experienced our clinical and radiological outcomes before their treatment switch. The different size of the study groups could be a partial limit of the first part of our study since RMS patients represent nearly 70% of the whole population. However, we think that this aspect did not significatively affect our results since the biomarkers’ concentration of both control groups and PMS group showed a very low SD and a normal distribution.

## Conclusions

In the present work, we demonstrated that the CSF CXCL13 and CHI3L1 levels, at the time of diagnostic evaluation, represent very good prognostic biomarkers in RMS patients and therefore can assist in the initial treatment choice. Patients with a higher concentration of both these proteins in CSF have a significantly higher risk of succeeding clinical and radiological activity. We also found that higher CSF concentrations of several neuro-inflammatory biomarkers are associated with a higher risk of conversion to CDMS in patients with a first clinical demyelinating event. Lastly, we found differential CSF BAFF and APRIL levels between RMS and PMS. This finding by further highlighting the role of B cells in MS pathology also suggest a differential modulation of B cell–related pathways in the different phases of the disease.

## Supplementary Information

Below is the link to the electronic supplementary material.

Supplementary file1 (DOCX 21 KB)

## Data Availability

The datasets generated during and/or analyzed during the current study are available from the corresponding author on reasonable request.
