# Supplementary material for: CSF CXCL13 and Chitinase 3-like-1 Levels Predict Disease Course in Relapsing Multiple Sclerosis
Source: Mol Neurobiol. 2022 Oct 10;60(1):36–50. doi: 10.1007/s12035-022-03060-6 (PMC9758105; doi:10.1007/s12035-022-03060-6)
Supplement: Supplementary file 1 — Supplementary file1 (DOCX 21 KB) [file 12035_2022_3060_MOESM1_ESM.docx]

**Supplementary figures**

**Supplementary Figure 1:** Correlation between CSF concentration of CXCL10 and disease severity parameters (Progression Index in A and MSSS in B) in PMS. (Spearman correlation r 0.61 and 0.74 respectively, p-value <0.01 in both cases). CSF: cerebrospinal fluid; PMS: Progressive Multiple Sclerosis.

**Supplementary Figure 2:** ROC curve to predict MS diagnosis following CSF levels of CHI3L1 (A), CHI3L1 after exclusion of OIND (B), CXCL13 (C), and CXCL13 after exclusion of OIND (D). p<0.01 for all analyses. CSF: cerebrospinal fluid. ROC: receiver operating characteristic.

**Supplementary Tables:**

**Supplementary Table 1.** OIND cohort composition.

| **OIND n=10** | **n** |
| --- | --- |
| CNS isolated vasculitis | 3 |
| Anti-phospholipid syndrome with brain MRI alterations | 3 |
| Systemic lupus erythematosus with neurological manifestations | 2 |
| Sjogren syndrome with neurological manifestations | 1 |
| Chronic inflammatory neuropathy | 1 |

*OIND: other inflammatory neurological disorders; CNS; Central Nervous System; MRI: Magnetic Resonance Imaging.*

**Supplementary Table 2.** First DMT in RMS patients.

| **Patients N=107** | **n (%)** |
| --- | --- |
| None | 8 (7.5) |
| Glatiramer acetate | 7 (6.5) |
| Interferon beta | 10 (9.3) |
| Teriflunomide | 8 (7.5) |
| Dimethyl fumarate | 46 (43.0) |
| Fingolimod | 12 (11.2) |
| Natalizumab | 10 (9.3) |
| Ocrelizumab | 4 (3.7) |
| Alemtuzumab | 2 (1.9) |

*DMT: disease-modifying therapy; RMS: Relapsing Multiple Sclerosis*

**Supplementary Table 3.** Conversion to CDMS.

| **Patients**  **N=88** | **Converting patients**  **N=29** | **Not converting patients**  **N=59** | **p value** |
| --- | --- | --- | --- |
| APRIL | 69.3 (46.4)  63.5 [26.8-111.6] | 54.2 (32.5)  57.2 [36.7-77.9] | 0.136 |
| BAFF | 154.8 (104.6)  104.6 [90.4-172.5] | 122.4 (49.1)  114.9 [80.9-158.6] | 0.407* |
| Chitinase 3 like 1, ng/ml | 542.6 (612.9)  239.1 [138.3-370.8] | 172.9 (223.8)  113.7 [84.6-174.0] | **<0.001*** |
| CCL2 | 310.0 (161.9)  261.9 [210.8-370.8] | 278.6 (116.4)  274.8 [180.5-353.3] | 0.622* |
| CXCL8 | 41.4 (22.0)  36.3 [28.4-43.4] | 40.0 (64.0)  30.3 [22.0-40.4] | 0.909 |
| CXCL10 | 137.1 (140.0)  95.2 [60.0-157.0] | 56.8 (37.7)  47.8 [28.9-73.9] | **<0.001*** |
| CXCL12 | 799.2 (410.0)  806.5 [485.3-1022.6] | 526.2 (330.4)  516.3 [199.2-749.5] | **<0.001** |
| CXCL13 | 38.8 (48.3)  19.9 [12.5-41.0] | 12.0 (8.3)  9.7 [7.3-13.6] | **<0.001*** |

*All values are reported as mean (standard deviation) in the first row and median [interquartile range] in the second row. In bold are reported significant differences at a two-sided α level <0.05. CDMS: clinically defined Multiple Sclerosis.*

**Supplementary Table 4.** Demographic, clinical, and radiological features of RMS grouped by CHI3L1 CSF concentration.

| **RMS Patients**  **N=105** | **CHI3L1 low concentration**  **N=36** | **CHI3L1 intermediate concentration N=42** | **CHI3L1 high concentration**  **N=29** | **p value** |
| --- | --- | --- | --- | --- |
| Female sex, n (%) | 30 (83.3) | 32 (76.2) | 18 (62.1) | 0.141 |
| Age, years | 34.5 (11.0) | 38.6 (10.4) | 39.3 (9.7) | 0.126 |
| OCBs, n (%) | 27 (75.0) | 34 (81.0) | 25 (86.2) | 0.524 |
| FUP duration (years) | 3.6 (1.3) | 3.5 (1.4) | 3.5 (1.5) | 0.271 |
| Disease Duration (months), median [IQR] | 3.5 [1.3-18.8] | 3.5 [2-27.3] | 4 [2-12] | 0.820 |
| EDSS, median [IQR] | 1.5 [1.0-2.0] | 1.5 [1.0-2.0] | 2.0 [1.5-2.5] | **0.002** |
| Gd+ lesion at baseline, n (%) | 17 (50.0) | 21 (53.8) | 15 (53.6) | 0.693 |
| Spinal lesion at baseline, n (%) | 28 (77.8) | 31 (73.8) | 25 (86.2) | 0.454 |
| MSSS | 3.9 (1.7) | 3.6 (2.0) | 4.3 (2.2) | **0.002** |
| Relapses one-year preceding CSF collection | 0.8 (0.4) | 0.9 (0.4) | 1.0 (0.4) | 0.115 |
| Relapse 30 days preceding CSF collection, n (%) | 11 (30.6) | 13 (31.0) | 12 (41.4) | 0.586 |

*All values are reported as mean (standard deviation) unless indicated otherwise. In bold are reported significant differences at a two-sided α level <0.05. RMS: Relapsing Multiple Sclerosis; OCBs: Oligoclonal Bands. FUP: follow-up; IQR interquartile range; EDSS: Expanded Disability Status Scale; Gd+: gadolinium-enhancing: MSSS: Multiple Sclerosis Severity Score; CSF: cerebrospinal fluid.*

**Supplementary Table 5.** Demographic, clinical, and radiological features of RMS grouped by CXCL13 CSF concentration.

| **RMS Patients**  **N=105** | **CXCL13 low concentration**  **N=32** | **CXCL13 intermediate concentration N=56** | **CXCL13 high concentration**  **N=19** | **p value** |
| --- | --- | --- | --- | --- |
| Female sex, n (%) | 29 (90.6) | 32 (69.6) | 12 (63.2) | **0.041** |
| Age, years | 39.9 (10.4) | 37.2 (10.8) | 33.9 (9.5) | 0.139 |
| OCBs, n (%) | 25 (78.1) | 45 (80.4) | 16 (84.2) | 0.869 |
| FUP duration (years) | 3.5 (1.5) | 3.3 (1.3) | 3.7 (1.3) | 0.085 |
| Disease Duration (months), median [IQR] | 6.5 [2-62] | 3 [1-8.8] | 3 [2-7] | 0.820 |
| EDSS, median [IQR] | 1.3 [1.0-2.0] | 1.5 [1.5-2.0] | 2.0 [1.0-2.5] | 0.228 |
| Gd+ lesion at baseline, n (%) | 10 (31.2) | 35 (64.8) | 8 (53.3) | **0.016** |
| Spinal lesion at baseline, n (%) | 26 (81.3) | 40 (71.4) | 18 (94.7) | 0.092 |
| MSSS | 3.1 (1.8) | 4.5 (2.0) | 5.0 (2.2) | **0.001** |
| Relapses one-year preceding CSF collection | 0.8 (0.4) | 1.0 (0.3) | 1.0 (0.5) | **0.030** |
| Relapse 30 days preceding CSF collection, n (%) | 8 (25.0) | 22 (39.3) | 6 (31.6) | 0.386 |

*All values are reported as mean (standard deviation) unless indicated otherwise. In bold are reported significant differences at a two-sided α level <0.05. RMS: Relapsing Multiple Sclerosis; OCBs: Oligoclonal Bands. FUP: follow-up; IQR interquartile range; EDSS: Expanded Disability Status Scale; Gd+: gadolinium-enhancing: MSSS: Multiple Sclerosis Severity Score; CSF: cerebrospinal fluid.*
